# Clinical and genetic characteristics of concomitant Mucopolysaccharidosis type IVA and neurogenic bladder in children: two case reports and literature review

**DOI:** 10.1186/s12887-020-02484-0

**Published:** 2021-01-06

**Authors:** Zhuhui Ge, Jianhua Mao, Huijun Shen, Yu Xu, Haidong Fu, Weiwei Zhang, Dongyan Li

**Affiliations:** 1 Department of Nephrology, the Children’s Hospital, Zhejiang University School of Medicine, 3333 Binsheng Road, Hangzhou, China; 2 Department of Pediatrics, the Frist Hospital of Ninghai, 142 Taoyuan Middle Road, Ninghai County, Zhejiang Province China; 3 Department of Hematology, the People’s Hospital of Ruian, 108 Wansong Road, Rui ’an City, Zhejiang Province China

**Keywords:** Mucopolysaccharidosis IVA, Neurogenic bladder, Pathogenesis, Comorbidity

## Abstract

**Background:**

Mucopolysaccharidosis IVA (MPS IVA; Morquio A syndrome) is a rare autosomal recessive lysosomal storage disorder. Up to now, reports on the clinical characteristics of MPS IVA mainly focused on patients with progressive bone dysplasia and multiple organ damage, while the effects of this disorder on neurogenic bladder have not been reported. Therefore, the aim of the present study is to report two cases of nocturnal enuresis finally diagnosed as neurogenic bladder in MPS IVA.

**Case presentation:**

Both children were characterized by the presence of pectus carinatum, kyphoscoliosis, nocturnal enuresis, urinary incontinence, normal intelligence, and loss of strength in the legs, diagnosed as neurogenic bladder in association with MPS IVA through the analysis of the clinical characteristics, enzyme activity and genetic testing. In addition, the terminator codon mutation c.1567T > G (p.X523E) and a novel missense mutation c.575A > G (p.E192G) were found in the coding region of the GALNS gene of the 1^st^ patient, while the missense mutation c.488C > A (p.P163H) was found in the coding region of the GALNS gene of the 2^nd^ patient.

**Conclusions:**

Neurogenic bladder may occur in patients with MPS IVA after spinal cord injury. It is necessary to screen for the diagnosis of MPS IVA in patients with atypical enuresis and skeletal abnormalities through the analysis of the clinical characteristics, enzyme activity and genetic testing.

## Background

Mucopolysaccharidosis (MPS) is a rare autosomal recessive inherited metabolic disease characterized by the deficiency of some enzymes in lysosomes required for the degradation of acidic mucopolysaccharides [1]. This disease features the deposition of mucopolysaccharides in cells, tissues and organs, resulting in damage to multiple organs and systems. MPS is classified into different subtypes according to the lack of different enzymes, and MPS IVA (also called Morquio A syndrome) is caused by the deficiency of N-acetylgalactosamine-6-sulfate sulfatase (GALNS). Deficiency of this enzyme leads to the accumulation of specific glycosaminoglycans (GAGs), keratin sulfate (KS) and chondroitin-6-sulfate (C6S), which are mainly produced and stored in cartilage [1]. Reports to date have demonstrated that some patients with MPS IVA experience severe systemic bone dysplasia, but that others may show only a mild form. The most common characteristics of MPS IVA are short stature, kyphoscoliosis, abnormal gait, laxity of the wrist joints, genu valgum, and pectus carinatum [2]. Other symptoms include restrictive lungs, cardiac complications, vision impairment, hearing impairment, and corneal clouding [1].

Despite numerous descriptions of these unique clinical characteristics, to the best of our knowledge, no reports are available on MPS IVA combined with neurogenic bladder. Here, we report two cases of nocturnal enuresis finally diagnosed as neurogenic bladder in MPS IVA.

## Case presentation

### Case 1

A 10-year-old male with nocturnal enuresis, considered the 1^st^ patient, was admitted to the paediatric department of our hospital on April 10, 2019. The 1^st^ patient did not have a history of birth asphyxia. This patient was born with a weight of 3.1 kg. His consanguineous parents were healthy, without a family history of inherited diseases. At the age of 6, the 1^st^ patient began showing weakness in both lower limbs, and progressive aggravation forced him to use a wheelchair. Despite having been trained to use the toilet since childhood, at the age of 9, the 1^st^ patient began to wet the bed with frequent urination, incontinence and delayed urination during the daytime. Physical examination revealed the following characteristics: weight of 30 kg and height of 140 cm (standard deviation of ± 1 in normal children of the same sex and age). He showed a clear mind and normal intelligence, with pectus carinatum, breast uplift, rib valgus, and poor mobility. Neurological investigation revealed sensory loss in the sacral area, muscle weakness, and decreased knee reflexes and plantar reflexes in the lower limbs. The anal reflex and cremasteric reflex were present. No other abnormal signs or symptoms were found, such as aberrant face, short stature, corneal clouding, impaired vision, impaired hearing, laxity of the wrist joints, genu valgum, or hepatosplenomegaly, which are typical of MPS IVA.

Echocardiography of the 1^st^ patient revealed tricuspid regurgitation. Ultrasonography of the urinary system revealed that both kidneys and ureters were normal without residual urine, the bladder capacity was 120 ml during the filling phase; the expected bladder capacity was 330 ml. Magnetic resonance spectroscopy (MRI) of the lumbar spine showed lumbar kyphosis at the L1-L2 level, stenosis of the spinal canal, disc protrusion, nerve compression due to cauda equina and a position inclining backward. The 1^st^ patient had a normal β-galactosidase enzyme activity of 278.24 nmol/(h.mg) [reference range 78.3-441.44 nmol/(h.mg)] and a very low leukocyte GALNS enzyme activity of 0.94 nmol/(17 h.mg) [reference range 24.44-216.69 nmol/(17 h.mg)] .

### Case 2

**Fig. 1:**
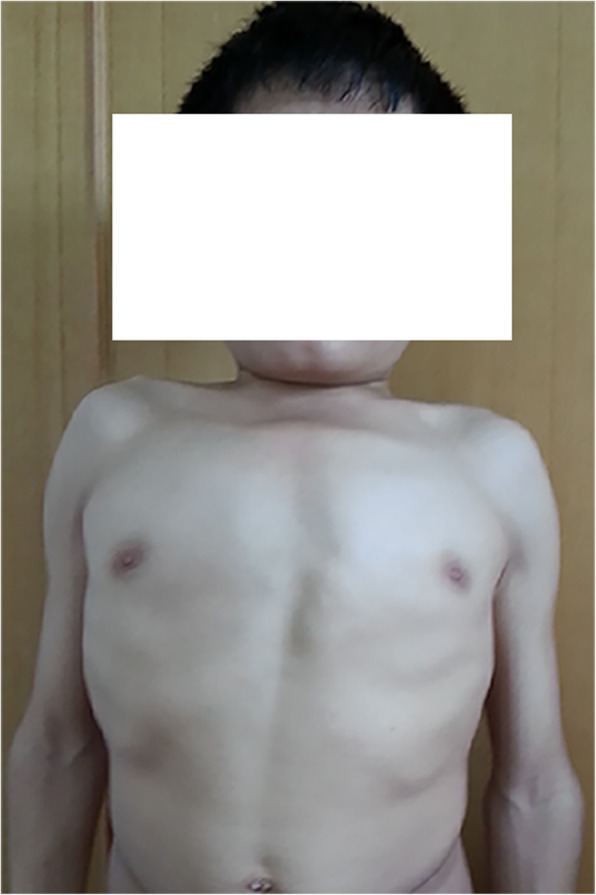
Images of the 2^nd^ patient with MPS IVA. The clinical image shows pectus carinatum, breast uplift and rib valgus

**Fig. 2:**
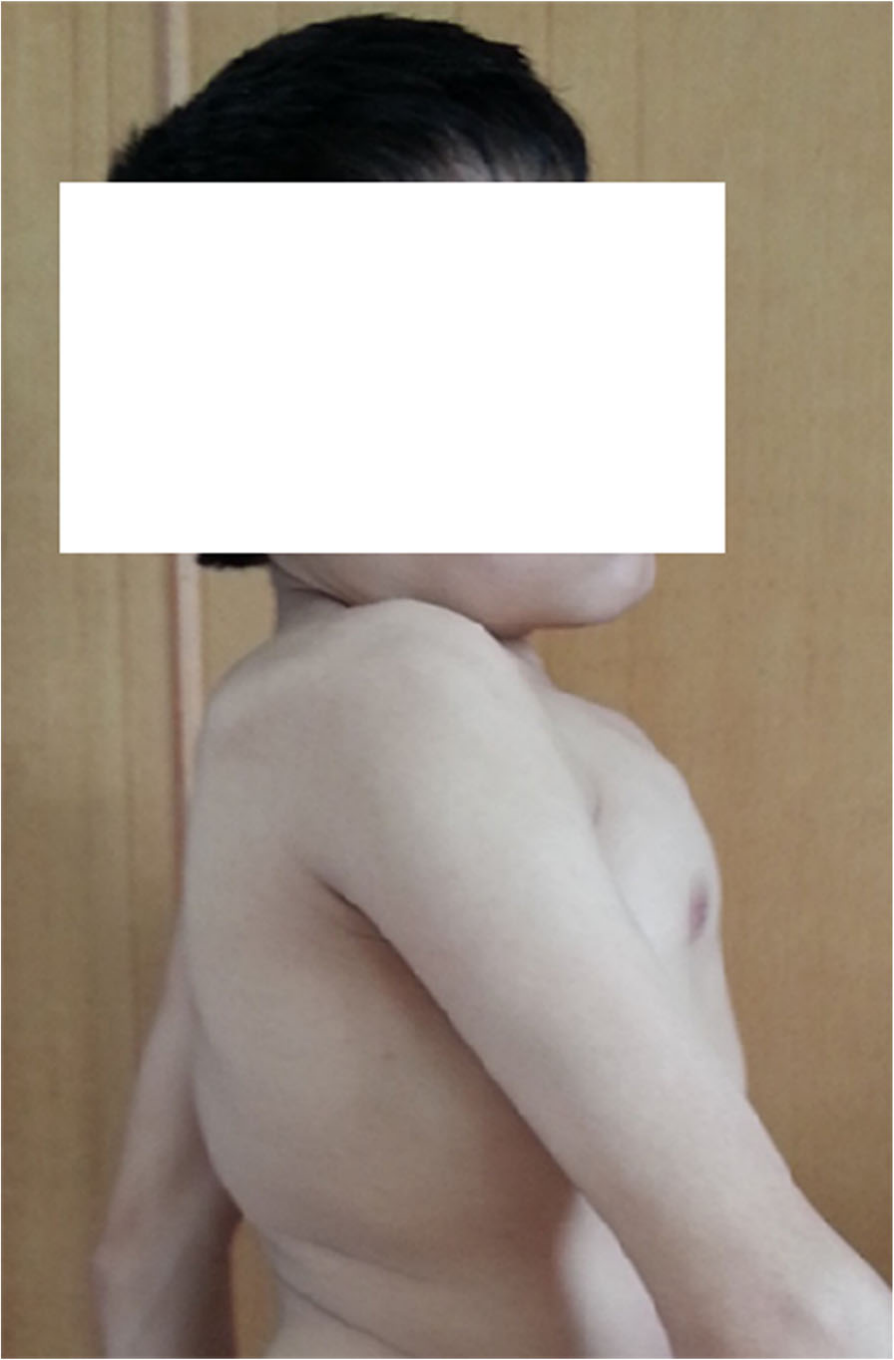
Images of the 2^nd^ patient with MPS IVA. The clinical image shows short stature, short neck and kyphoscoliosis

An 18-year-old male, considered the 2^nd^ patient, went to several hospitals with a complaint of bed wetting over the previous 15 years, and nocturnal enuresis was suspected. On April 3, 2014, the 2^nd^ patient was transferred to the paediatric outpatient department of our hospital. The 2^nd^ patient was a first-born child, and he did not have a history of birth asphyxia; his consanguineous parents were healthy, without a family history of inherited diseases. At the age of 3, although he was trained to use the toilet, he began to wet the bed, accompanied by frequent urination, urinary incontinence, progressive weakness in both lower limbs, and a waddling gait. At the age of 14, nocturnal enuresis became more remarkable, and he was not able to walk without support. Based on physical examination, the 2^nd^ patient had a weight of 50 kg and a height of 152 cm (below the standard deviation of -3 SD in normal children of the same sex and age). He showed normal intelligence, with no deformed face, short neck, pectus carinatum, abnormal gait, kyphoscoliosis, breast uplift or rib valgus (Figs. 1 and 2). Neurological investigation revealed sensory loss in the saddle region, loss of knee reflexes and plantar reflexes in the lower limbs, decreased anal reflex and cremasteric reflex, and motor weakness resulting in walking difficulty. No other abnormal symptoms or signs typical of MPS IVA were found, such as corneal clouding, impaired vision, impaired hearing, laxity of the wrist joints, genu valgum, or hepatosplenomegaly.

**Fig. 3:**
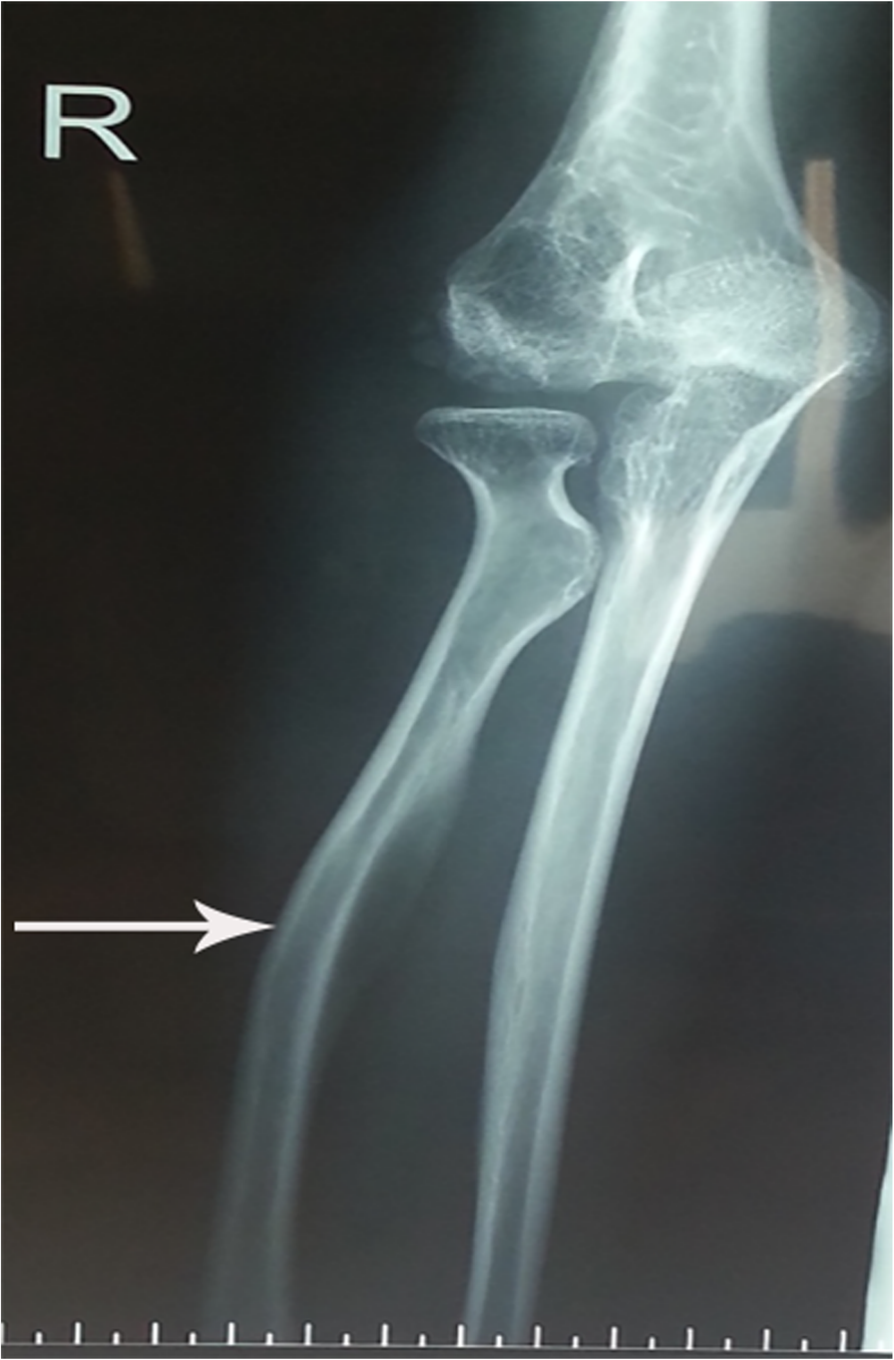
The X-ray radiograph of the 2^nd^ patient shows that the distal radius was tilted towards the ulna

**Fig. 4:**
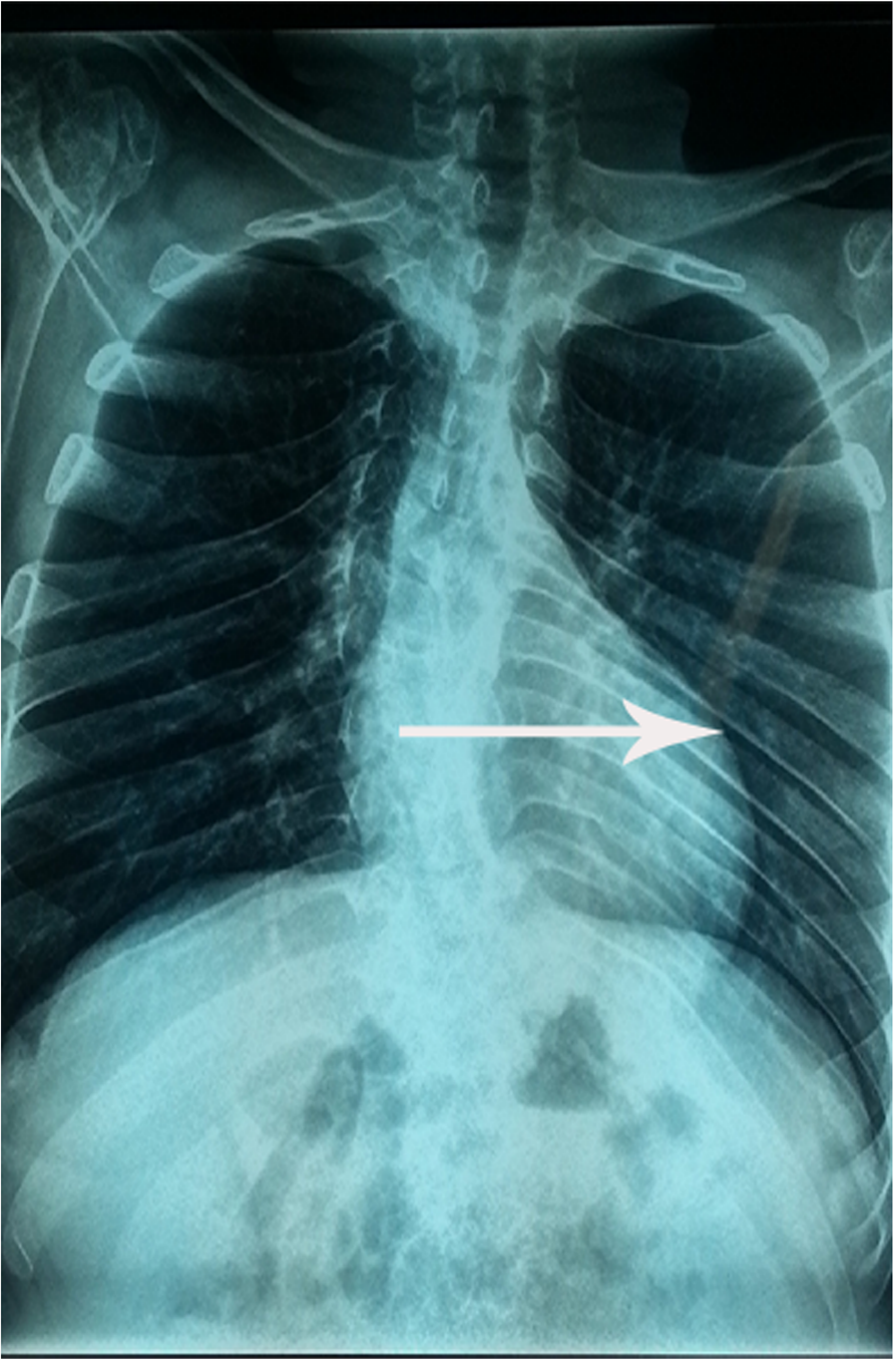
The X-ray radiograph of the spine of the 2^nd^ patient shows scoliosis and characteristic oar-shaped ribs

Based on ultrasonography of the urinary system, the size of the left kidney was 10.5 cm × 6.8 cm, and the size of the right kidney was 10.2 cm × 6.5 cm. The bladder size was 14.2 cm × 8.5 cm × 6.8 cm during the filling phase, and the bladder wall was thickened (1.12 cm thick) and rough. After the release of 80 ml of urine, the bladder size was 14.2 cm × 7.6 cm × 5.9 cm, and the thickness of the bladder wall was 1 cm. A plain film X-ray revealed lateral curvature of the thoracolumbar spine, flattening and irregular edges of the vertebral body, characteristic oar-shaped ribs, and the distal radius tilting towards the ulna (Figs. 3 and 4). MRI of the lumbar spine revealed lumbosacral kyphosis at the L2-L3, L3-L4, L4-L5 and L5-S1 levels, disc protrusion and cauda equina compression.

### GALNS gene mutation analysis

According to the American College of Medical Genetics and Genomics (ACMG) guidelines, three different mutations were identified in these two children in the present study: 2 missense and 1 terminator codon mutation (in Table 1). Sequence analysis of the GALNS gene in the 1^st^ patient led to the identification of a known mutation and a new alteration. The known mutation c.1567T > G (p.X523E) changes the termination codon to glutamic acid, which extends the peptide chain by 92 amino acids, and the terminator codon mutation is a pathogenic mutation [3]. The new alteration c.575A > G (p.E192G) is a novel missense mutation that is also a potential pathogenic mutation, and two heterozygous mutations were identified in the 1^st^ patient. All mutations were confirmed using the Sanger sequencing method for pedigree validation, and all mutations were inherited from his parents. The results of GALNS gene mutation analysis in the 2^nd^ patient revealed a homozygous mutation. The missense mutation c.488C > A (p.P163H) leads to significant changes in the structure of the protein [4].

**Table 1:** Clinical characteristics and results of the analysis of GALNS gene mutation in the two patients

Gene	Patient	Sex	Type of Mutation	Nucleotide	Protein	Zygosity	Segregation	Age of onset (years)	ACMG hazard level	Ref
GALNS	1^st^	M	Missense	c.575A>G;	p.E192G	het	m		Likely pathogenic	ND
								6		
			Terminator	c.1567T>G;	p.X523E	het	f		Pathogenic	[3]
GALNS	2^nd^	M	Missense	c.488C>A;	p.P163H	hom	ND	3	Likely pathogenic	[4]

*ACMG* According to the American College of Medical Genetics and Genomics guidelines, *Ref* Reference, *het* Heterozygous, *hom* Homozygous, *M* Male, *m* Mother, *f* Father, *ND* No data or not done

### Outcome and follow-up

Regarding the 1^st^ patient, the paediatric neurosurgeon recommended a complete preoperative examination before deciding to perform lumbar dilated spinal cord decompression, but his family members preferred a period of observation time and refused consent for the operation.

Regarding the 2^nd^ patient, the paediatric nephrologists suggested first adopting conservative treatment for symptom improvement, such as the administration of oral desmopressin tablets (0.2 mg/night) and tolterodine tartrate tablets (2 mg/day), and clean intermittent catheterization, with continued outpatient follow-up once a month.

However, due to the lack of drugs for effective enzyme replacement therapy, the two patients did not show any significant improvement in symptoms during the follow-up.

## Discussion and conclusions

MPS IVA is an autosomal recessive disorder caused by a lack of the GALNS enzyme. As mentioned above, deficiency of this enzyme leads to the accumulation of specific GAGs, KS and C6S, which are mainly produced and stored in cartilage, resulting in a direct impact on cartilage and bone development and subsequent unique systemic skeletal dysplasia [1]. Furthermore, the incomplete and subsequent growth imbalance becomes more evident with advancing age [1]. Without proper treatment, patients with the severe form of MPS IVA generally do not survive beyond 30 years; patients with mild MPS IVA are not able to survive beyond 70 years [5]. Therefore, early diagnosis and treatment are helpful to improve quality of life. Nevertheless, as the clinical symptoms of MPS IVA overlap with those of other MPS types, diagnosis of MPS IVA based on clinical characteristics is very difficult.

The clinical features of MPS involve multiple organ damage, in addition to bone deformation and multiple bone dysplasia revealed by X-ray. Indeed, MPS can also affect the central nervous system, cardiovascular system, liver, spleen, joints, tendons and skin. Although the different MPS types have many similar clinical characteristics, severe skeletal malformations and normal intelligence represent the main difference between MPS IVA and other MPS types [6]. Many of the symptoms of MPS IVA have been reported, but a detailed description of the effects of mucopolysaccharide metabolism disorder on neurogenic bladder in association with MPS IVA is not available. In the present study, both patients presented lower limb weakness and nocturnal enuresis, accompanied by daytime symptoms of the lower urinary tract such as urgency, incontinence, and delayed urination, which should be considered non-monosymptomatic enuresis [7]. MRI of the lumbar spine in the 1^st^ patient revealed lumbar kyphosis at the L1-L2 level, stenosis of the spinal canal, disc protrusion, and nerve compression. Compared with the expected bladder capacity, ultrasonography of the urinary system revealed a smaller capacity during the filling phase. Urinary system ultrasound of the 2^nd^ patient indicated a large bladder capacity and residual urine in the bladder, and MRI of the lumbar spine showed lumbosacral kyphosis at the L2-L3, L3-L4, L4-L5 and L5-S1 levels, disc protrusion and cauda equina compression. Accordingly, based on the results of the clinical characteristics, neurological findings and lumbar spine MRI, the diagnosis in both patients was neurologic bladder caused by lumbosacral dysplasia and spinal cord injury. Neurogenic bladder dysfunction is caused by injury to the neural pathways (including the central nervous system, peripheral nervous system and neuromuscular junctions) that regulate and control lower urinary functions[8]. Regarding the peripheral nervous system, parasympathetic innervation (S2-S4) can excite the smooth muscle of the bladder and inhibit urethral sphincter smooth muscle; sympathetic innervation (T10-L2) can excite the bladder neck and inhibit bladder smooth muscle [8]. This view is also consistent with the results of the present study. Overall, urodynamic analyses to assess detrusor and sphincter function, bladder pressure, and bladder volume are important examinations in the management of neurogenic bladder patients. Nevertheless, as the two patients were unwilling to undergo urodynamic examination, further urodynamic results are not available. In a recent study, Remondino et al. [9] reported 52 MPS patients with clinical manifestations of spinal lesions, such as cervical spine involvement (43 patients), thoracolumbar kyphosis (14 patients), spinal cord compression (8 patients), and stenosis of the spinal canal (7 patients); among all patients, 55% showed nerve damage. As a result, annual MRI of the whole spine is recommended in patients with MPS IVA to assess spinal disorders [10]. Additionally, Budak et al. [11] considered that urinary GAG excretion may be an important marker for bladder injury and the cause of bladder wall degeneration in patients with neurogenic bladder may be that the bladder wall becomes enriched in GAGs. Indeed, they measured GAG excretion in 20 patients with primary nocturnal enuresis, 43 patients affected by spina bifida and a control group, and the results revealed significantly higher urinary GAG excretion in the patients than in the control group [11, 12]. Because of the small sample size, further analysis should be performed to confirm this finding.

It is a challenge to correctly diagnose MPS IVA, especially in its attenuated form, because of the wide spectrum of clinical characteristics in patients with MPS IVA and because the radiographic findings are similar to those in patients with multiple epiphyseal dysplasia. Experts have presented a diagnostic algorithm based on the decline in growth velocity as well as bone and joint involvement that was designed to help paediatricians identify the early characteristics of the attenuated forms of MPS [13]. Once MPS is clinically suspected, urinary GAG analysis and enzyme detection are crucial for accurate diagnosis and prognosis of MPS subtypes [13]. However, the clinical features in patients with mild MPS are atypical, and mutation in other genes can lead to decreased GALNS activity such as multiple sulfatase deficiency, increasing the difficulty of diagnosing this disease [14]. In recent years, the study of molecular genetics and metabolic diseases by Chinese and scientists abroad has greatly improved genetic diagnosis. The GLANS gene located on chromosome 16q24.3 contains 14 exons and encodes a 1566-bp cDNA [15]. As of February 2018, more than 334 mutations in the GALNS gene had been reported: 203 missense/nonsense mutations, 35 deletions, 22 splicing site mutations, 7 insertions and 3 complex rearrangements [1]. Several GALNS gene mutations are common, such as c.1156C > T (p.R386C), c.901G > T (p.G301C), c.337A > T (p.I113F), c.1A > G (p.M1V), and c.757C > T (p.R253W), accounting for 8.9%, 6.8%, 5.7%, 2.3%, and 2.1% of the mutations studied, respectively [1, 16–19]. In the present study, the 1^st^ patient carried the compound heterozygous mutation c.1567T > G (p.X523E) and c.575A > G (p.E192G). In addition, two heterozygous mutations were present in the parents. The 2^nd^ patient harboured a homozygous mutation c.488C > A (p.P163H) in the GALNS gene. Both showed kyphoscoliosis and nocturnal enuresis and urinary incontinence. The 2^nd^ patient with the homozygous mutation had an earlier onset of symptoms that were also more evident than those of the 1^st^ patient. In another Chinese study, 12 different mutations were identified in 9 MPS IVA patients from southern China, including 3 novel missense mutations, and all patients showed varying degrees of thoracolumbar spine kyphosis [20], though symptoms of neurogenic bladder were not described. However, a case of neurogenic bladder was reported in 1994 in patients with other types of MPS, and Koyama et al. reported a rare case of a patient with Hunter’s syndrome and neurogenic bladder [21].

At present, effective treatments for MPS IVA are enzyme replacement therapy and allogeneic haematopoietic stem cell transplantation (allo-HSCT). Other treatments are limited to palliative care and alleviating symptoms [20]. In general, progression of functional impairment is an indication of compression in the spinal cord of MPS patients; thus, it is an indication that surgery is needed, and early spinal decompression may prevent or reverse neurologic impairment [9]. Regardless, treatment to block neurogenic bladder in MPS IVA patients has never been reported. In the present study, decompression of the spinal cord was recommended for the 1^st^ patient who had evidence of spinal cord compression; however, the family was concerned about the recovery process after the operation. In contrast, conservative treatment was recommended for the 2^nd^ patient, but follow-up revealed the ineffectiveness of these drugs, as enzyme replacement drugs were not available in China until 2019. In 2014, the Federal Drug Administration (FDA) of the USA approved BioMarin Pharmaceutical elosulfase alfa (trade name Vimizim injection) for the treatment of MPS IVA [22], and the National Medical Products Administration (NMPA) also approved it in 2019. Elosulfase alfa is effective in increasing the walking distance and improving the respiratory function of patients [23]. In another study from Taiwan, six MPS IVA patients received 4-6.5 years of enzyme replacement therapy, such as a weekly intravenous injection of Elosulfase alfa (2.0 mg/kg), and the results showed that biochemical and clinical characteristics were improved [24]. Nonetheless, some limitations are that the enzyme cannot cross the blood-brain barrier; thus, it has an insufficient effect on symptoms of the central nervous system. In addition, enzyme replacement therapy is expensive and should be administered for a lifetime. In general, allo-HSCT can enable patients with MPS IVA to achieve permanent enzyme production. Macrophages of healthy donors can pass through the blood-brain barrier and partially alleviate symptoms of the central nervous system [25, 26], and a long-term study on enzyme replacement therapy and allo-HSCT indicated some improvement in symptoms and daily living activity [5, 24]. If patients are promptly treated with enzyme replacement therapy or allo-HSCT at the time of symptom onset, these therapies might partially improve neurogenic bladder because many of the organ dysfunctions in MPS IVA are progressive; it should be noted that it is still unknown whether these symptoms disappear. A study on the development of gene therapy for MPS IVA has recently been reported, involving a possible one-time permanent treatment [5].

Overall, the cases described in the present study revealed that neurogenic bladder might occur in patients with MPS IVA after spinal cord injury. Therefore, special attention should be paid to the analysis of clinical characteristics, enzyme activity and genetic testing in children with atypical enuresis to exclude the possibility of potential MPS IVA. Although this is the first report of neurogenic bladder as a symptom of MPS IVA, further studies should be performed to confirm the existence of other patients with this symptom to confirm it as another characteristic of MPS IVA.

## Data Availability

The data that support the findings of this study are available from the corresponding author upon reasonable request.

## Abbreviations

MPS IVA: Mucopolysaccharidosis IVA
MPS: Mucopolysaccharidosis
GALNS: N-acetylgalactosamine-6-sulfate sulfatase
GAGs: Glycosaminoglycans
C6S: Chondroitin-6-sulfate
MRI: Magnetic resonance spectroscopy
ACMG: the American College of Medical Genetics and Genomics
allo-HSCT: Allogeneic hematopoietic stem cell transplantation
FDA: Federal Drug Administration
NMPA: National Medical Products Administration

## References

[1] Peracha H, Sawamoto K, Averill L, Kecskemethy H, Theroux M, Thacker M (2018). Molecular genetics and metabolism, special edition: Diagnosis, diagnosis and prognosis of Mucopolysaccharidosis IVA. Mol Genet Metab.

[2] Hendriksz CJ, Harmatz P, Beck M, Jones S, Wood T, Lachman R (2013). Review of clinical presentation and diagnosis of mucopolysaccharidosis IVA. Mol Genet Metab.

[3] Dung VC, Tomatsu S, Montano AM, Gottesman G, Bober MB, Mackenzie W (2013). Mucopolysaccharidosis IVA: correlation between genotype, phenotype and keratan sulfate levels. Mol Genet Metab.

[4] Wang Z, Zhang W, Wang Y, Meng Y, Su L, Shi H (2010). Mucopolysaccharidosis IVA mutations in Chinese patients: 16 novel mutations. J Hum Genet.

[5] Khan S, Almeciga-Diaz CJ, Sawamoto K, Mackenzie WG, Theroux MC, Pizarro C (2017). Mucopolysaccharidosis IVA and glycosaminoglycans. Mol Genet Metab.

[6] Suarez-Guerrero JL, Gomez HP, Arias FJ, Contreras-Garcia GA (2016). [Mucopolysaccharidosis: clinical features, diagnosis and management]. Rev Chil Pediatr.

[7] Wang T, Yang SS, Tsai J, Yu M, Chiou Y, Chen K (2019). Management of nocturnal enuresis in Taiwan: Consensus statements of the Taiwan enuresis expert committee. J Formos Med Assoc.

[8] Samson G, Cardenas DD (2007). Neurogenic bladder in spinal cord injury[J]. Phys Med Rehabil Clin N Am.

[9] Remondino RG, Tello CA, Noel M, Wilson AF, Galaretto E, Bersusky E (2019). Clinical Manifestations and Surgical Management of Spinal Lesions in Patients With Mucopolysaccharidosis: A Report of 52 Cases. Spine Deform.

[10] Akyol MU, Alden TD, Amartino H, Ashworth J, Belani K, Berger KI (2019). Recommendations for the management of MPS IVA: systematic evidence and consensus-based guidance. Orphanet J Rare Dis.

[11] Budak YU, Huysal K, Guray A (2010). Urinary glycosaminoglycan excretion in patients with primary nocturnal enuresis. Ital J Pediatr.

[12] Salvaggio E, Antuzzi D, Ferrara P (2001). Glycosaminoglycans: urinary excretion in children with myelomeningocele[J]. Urol Int.

[13] Guffon N, Journeau P, Brassier A, Leger J, Chevallier B (2019). Growth impairment and limited range of joint motion in children should raise suspicion of an attenuated form of mucopolysaccharidosis: expert opinion. Eur J Pediatr.

[14] Morris CP, Guo XH, Apostolou S, Hopwood JJ, Scott HS (1994). Morquio A syndrome: cloning, sequence, and structure of the human N-acetylgalactosamine 6-sulfatase (GALNS) gene. Genomics.

[15] Baker E, Guo XH, Orsborn AM, Sutherland GR, Callen DF, Hopwood JJ (1993). The morquio A syndrome (mucopolysaccharidosis IVA) gene maps to 16q24.3. Am J Hum Genet.

[16] Montano AM, Kaitila I, Sukegawa K, Tomatsu S, Kato Z, Nakamura H (2003). Mucopolysaccharidosis IVA: characterization of a common mutation found in Finnish patients with attenuated phenotype. Hum Genet.

[17] Tomatsu S, Dieter T, Schwartz IV, Sarmient P, Giugliani R, Barrera LA (2004). Identification of a common mutation in mucopolysaccharidosis IVA: correlation among genotype, phenotype, and keratan sulfate. J Hum Genet.

[18] Tomatsu S, Filocamo M, Orii KO, Sly WS, Gutierrez MA, Nishioka T (2004). Mucopolysaccharidosis IVA (Morquio A): identification of novel common mutations in the N-acetylgalactosamine-6-sulfate sulfatase (GALNS) gene in Italian patients. Hum Mutat.

[19] Tomatsu S, Nishioka T, Montano AM, Gutierrez MA, Pena OS, Orii KO (2004). Mucopolysaccharidosis IVA: identification of mutations and methylation study in GALNS gene. J Med Genet.

[20] Xie J, Pan J, Guo D, Pan W, Li R, Guo C (2019). Mutation analysis and pathogenicity identification of Mucopolysaccharidosis type IVA in 8 south China families. Gene.

[21] Koyama K, Moda Y, Sone A (1994). Neurogenic bladder in Hunter’s syndrome[J]. J Med Genet.

[22] Sanford M, Lo JH (2014). Elosulfase alfa: first global approval. Drugs.

[23] Hendriksz CJ (2016). Elosulfase alfa (BMN 110) for the treatment of mucopolysaccharidosis IVA (Morquio A Syndrome). Expert Rev Clin Pharmacol.

[24] Lin HY, Chuang CK, Ke YY, Hsu CC, Chiu PC, Niu DM, et al.Long-term effects of enzyme replacement therapy for Taiwanese patients with mucopolysaccharidosis IVA. Pediatr Neonatol. 2018. 10.1016/j.pedneo.2018.08.005. 10.1016/j.pedneo.2018.08.005 30241882

[25] Turbeville S, Nicely H, Rizzo JD, Pedersen TL, Orchard PJ, Horwitz ME (2011). Clinical outcomes following hematopoietic stem cell transplantation for the treatment of mucopolysaccharidosis VI. Mol Genet Metab.

[26] Wang J, Luan Z, Jiang H, Fang J, Qin M, Lee V (2016). Allogeneic Hematopoietic Stem Cell Transplantation in Thirty-Four Pediatric Cases of Mucopolysaccharidosis—A Ten-Year Report from the China Children Transplant Group. Biol Blood Marrow Tr.

